# Prevalence and prognosis of molecular phenotypes in breast cancer patients by age: a population-based retrospective cohort study in western Algeria

**DOI:** 10.11604/pamj.2021.38.88.21370

**Published:** 2021-01-27

**Authors:** Amina Belhadj, Sonia Seddiki, Adel Belhadj, Badra Zakmout, Abd El Kader Amine Araba, Tewfik Sahraoui

**Affiliations:** 1 Biology of Development and Differentiation Laboratory, Oran 1 University, Ahmed Ben Bella, Oran, Algeria,; 2 Faculty of Medicine, Oran 1 University, Ahmed Ben Bella, Oran, Algeria,; 3 Department of Biology, Djillali Lyabes University, Sidi Bel Abbes, Algeria

**Keywords:** Breast, cancer, molecular phenotypes, age, prevalence, prognosis

## Abstract

**Introduction:**

breast cancer is related to age. The young age remains a controversial issue as a prognostic factor and have more aggressive clinical behavior with poor outcome. We aimed for the first time in Algeria to explore on a large cohort of patients the prevalence of the molecular phenotypes and to describe their clinical characteristics and survival.

**Methods:**

medical record of 1140 Algerian patients were analysed and categorized into three age groups: “young” when women were aged below 40 years; “middle-age” when women were aged from 41 to 54 years old and “elder” when women were over 54 years. Baseline categorical variables were analysed using the Chi-square test and survival curves were constructed using Kaplan Meir method.

**Results:**

the distribution of the various prognostic factors did not differ significativelly by age groups except for histological types, hormone receptors status and molecular phenotypes. Most patients were luminal A, indeed, young and intermediate age patients were most likely to be luminal A whereas the aged patients were triple negative with the highest mean DFS. Elsewhere young women are considered as human epidermal growth factor receptor 2 (HER2+) or triple negative molecular subtypes involving more rigorous therapeutic monitoring. The high rate of triple negative breast cancer in aged patients may due to genetic predispositions.

**Conclusion:**

this study sheds light on the histoclinical and molecular characteristics of breast cancer in young patients, which has a good prognosis than their older counterparts. Our results are therefore surprisingly different from what the literature suggests. A further study should understand this uncommon finding.

## Introduction

Breast cancer (BC) is the most frequently diagnosed cancer and the second most common cause of cancer death, in females worldwide [1]. In Arab countries BC occurs in younger women unlike western countries [2]. North African female breast cancer incidence seems to be 2-4 times lower than in western countries, BC accounts for 25% to 35% of all cancers with incidence between 16/100.00 inhabitants in Benghazi (Libya) (2003-2005) and 39/100,000 in Rabat (Morocco) in 2005 whereas in Sfax (Tunisia) the incidence was 25.2/100,000 inhabitants (2000-2002) [3].

In Algeria, BC is the leading cause of death among women with an incidence of 20.5/100,000 inhabitants in Setif between 2003-2007 and 35.2/100,000 inhabitants in Annaba between 2007 and 2009 [3] and 36.5/100,000 inhabitants in Oran between 1996 and 2011 [4], 11% of breast cancer cases occur in young women (≤35 years old) and 55% of cases at ≤50 years [5].

BC in young women (under 35 or 40) is uncommon, giving rise to considerable interest because of the unfavourable prognosis [6]. Young women with BC are characterized by less hormone sensitivity, higher human epidermal growth factor receptor 2 expressions and aggressive clinical behavior with poor outcomes as compared to the elderly group [7]. The number of elderly breast cancer patients is increasing and knowledge about possible differences in the biology and clinical outcomes of breast cancer according to age is limited even age is an important determinant of therapy [8]. Clearly, more information about biological and clinical features of BC is needed to support the different therapy approaches. In this study, we have, for the first time in Algeria, explored on a large series of patients, molecular phenotypes and clinical characteristics of different age groups of women diagnosed with BC, in order to describe prognosis factors association in relation with their respective ages at diagnosis.

## Methods

A total of 1140 female cases diagnosed as BC and underwent mastectomy from year 2008 to 2016 were retrospectively reviewed with regards to their histoloclinical and molecular features. Data were collected from pathological and oncological reports from three main west Algerian hospitals: public hospital of Oran, Military Hospital of Oran and public hospital of Sidi Bel Abbes. Exclusion criteria concerns women with other disease than BC, cases when HER2 was defined score 2 and male breast cancers. Ethical clearance from the institutional ethical committee was obtained.

For the analysis of breast cancer features, women were categorized into three age groups: younger than 40 years; middle-age, 44 to 54 years; and elderly, over 54 years. All data including pathological type, Scarff-Bloom-Richardson (SBR) grade, Tumor-Node-Metastasis (TNM) stages, hormone recepetors (HR) status, HER2 and Ki67 status and molecular phenotypes were compared. Breast cancer molecular phenotypes were defined basing on hormone receptors and HER2 status as follow: luminal A (ER+, PR+/-, HER2-), luminal B (ER+, PR+/-, HER2+), triple negative BC: TNBC (ER-, PR-, HER2-), HER2+ (ER-, PR-, HER2+).

Statistical analysis was performed by SPSS software package version 20.0 for windows (SPSS Inc., Chicago, USA). Quantitative data are presented as median ± standard deviation while the distribution of bio-pathological characteristics between groups was compared by means of the Chi-square test. Disease free survival (DFS) curves were estimated using the Kaplan-Meier method and statistical significance of pairwise comparisons was assessed using the log-rank test. DFS follow-up times were measured from the date of diagnosis till the first detection of breast cancer specific relapse or to the end of the study P-values <0.05 were considered significant.

## Results

Characteristics for all 1140 tumors are shown in Table 1. The median age of study population at the time of diagnosis was 50 years (range, 21-88 years) ±11.8 years. Among these, 195(17%) were <40 years (median age=37±4.10), 512 (45%) were aged between 40 and 54 years (median age=47±4.08) and 433 (38%) were 55 years and over (median age=62±6.70).

**Table 1:** distribution of pathologic features and molecular phenotypes of general population by age groups

Histological/phenotypes characteristics		Number of patients (%)	<40	(40-54)	≥55	P-Value
Histological types	Ductal	877 (79.4%)	154 (81.9%)	402 (80.7%)	321 (76.6%)	0.011
	Lobular	181 (16.4%)	20 (10.6%)	82 (16.5%)	79 (18.9%)	
	Other	47 (4.2%)	14 (7.4%)	14 (2.8%)	19 (4.5%)	
Inflammatory breast cancer	IBC	47 (5.3%)	6 (3.8%)	21 (5.4%)	20 (6%)	0.616
	NIBC	834 (94.7%)	150 (96.2)	370 (94.6 %)	176 (%)	
TNM staging	pT1	114 (13%)	19 (12.2%)	51 (13%)	44 (13%)	0.768
	pT2	449 (51%)	75 (48.5%)	198 (50.4%)	176 (52.4%)	
	pT3	142 (16%)	32 (20.6%)	61 (15.5%)	49 (14.6%)	
	pT4	179 (20%)	29 (18.7%)	83 (21.1%)	67 (20%)	
	M1 at the moment of diagnostic	82 (7.2%)	15 (7.6%)	38 (7.4%)	29 (6.6%)	0.007
Nodal status	N-	350 (35.3%)	61 (36.1%)	143 (32.4%)	146 (38.3%)	0.206
	N+	641 (64.7%)	108 (63.9%)	298 (67.6%)	235 (61.7%)	
SBR grade	I	38 (3.6%)	10 (5.6%)	14 (2.9%)	14 (3.5%)	0.269
	II	610 (57.5%)	98 (54.5%)	283 (59.2%)	229 (57%)	
	III	412 (38.9%)	72 (40%)	181 (37.9%)	159 (39.5%)	
Hormone receptors status	HR-	417 (39.1%)	60 (33.1%)	182 (37.7%)	175 (43.5%)	0.040
	HR+	649 (60.9%)	121 (66.9%)	301 (62.3%)	227 (56.5%)	
HER2 status	HER2-	714 (67.6%)	123 (68%)	313 (65.5%)	278 (69.8%)	0.358
	HER2+	343 (32.4%)	58 (32%)	165 (34.5%)	120 (30.2%)	
Ki 67 status	Ki 67 <14	230 (40%)	41 (45.1%)	93 (45.8%)	96 (45.1%)	0.986
	Ki 67 ≥ 14	277 (60%)	50 (54.9%)	110 (54.2%)	117 (54.9%)	
Molecular phenotypes	Luminal A	337 (31.6%)	69 (38.1%)	156 (32.2%)	112 (27.9%)	0.025
	Luminal B	313 (29.4%)	52 (28.7%)	146 (30.2%)	115 (28.7%)	
	HER2+	130 (12%)	18 (9.9%)	68 (14%)	44 (11%)	
	TNBC	286 (27%)	42 (23.2%)	114 (23.6%)	130 (32.4%)	

Out of the total participants, ductal carcinoma was the most frequent histological type (79.4%), followed by lobular carcinoma in 16.4% of cases, the other types of breast cancer are relatively uncommon including epidermoid carcinoma, meddulary carcinoma and phyllodes tumors in 4.2% of cases, thus, a significant difference between the age groups in the distribution of the histological types was found (P=0.011), with a slightly higher frequencies (81.9% and 80.7%) of ductal carcinoma in young and middle age groups respectively. Inflammatory breast cancer (IBC) is a rare and very aggressive disease in which cancer cells block lymph vessels in the skin of the breast. In the present study IBC tends to be more present among middle aged and older patients (5.4% and 6% respectively) with no statistical significance. Alternatively, we noted the predominance of SBR grade II (57.5%) compared to SBR III (38.9%) and patients were most hormone positive (60.9%), the lack of HER2 was observed in 67.6% of cases.

There was a higher incidence in node positive cases among middle aged patients, although this difference was not statistically significant. Tumor size is defined by measuring the tumor during pathological assessment in at least two dimensions, with the greatest dimension used for tumor staging (TNM staging), at the time of diagnosis most common T stage in the three groups was T2 (2cm).

The proportion of tumors with positive HR increased from 56.6% in patients 55 years and above to 62.3% in intermediate group to 68% in younger patients (below 40 years) (P=0.040). No difference in the overexpression of HER2/neu was observed between the three age groups (Table 1). The distribution of “intrinsic” breast cancer subtypes (basing on HR and HER2 status) in our cohort revealed that most patients were luminal A (31.6%) and a significant trend was noted when comparing the three age groups (P=0.025), indeed, young and intermediate age patients were most likely to be luminal A subtype whereas the aged patients (over 55 years) seems to have a bad prognostic since they presented a highest proportion of triple negative BC´s (TNBC´s) phenotype as indicated in Table 1.

**Figure 1:**
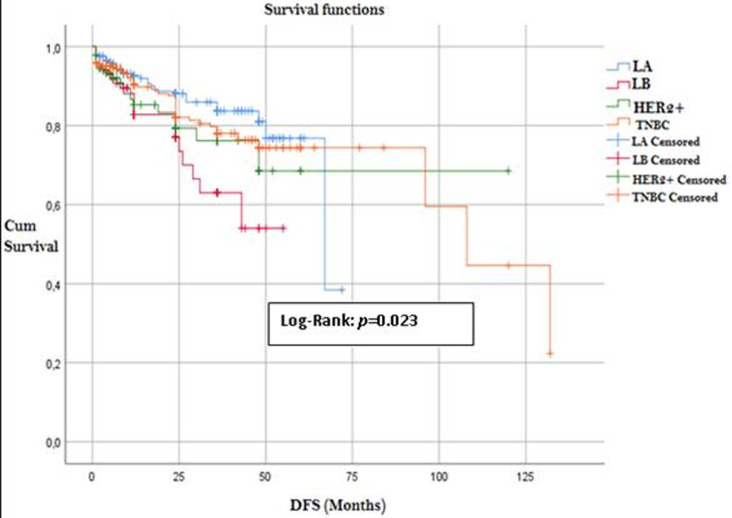
disease free survival among molecular breast cancer subtypes (Kaplan Meier)

Of the 1140 patients, 374 were excluded from the survival analysis for missing data. This resulted in a final sample of 766 patients in whom breast cancer was diagnosed for the first time between 2008 and 2014. Univariate analysis showed significant differences in survival between the molecular subtypes (P=0.023), individuals with TNBC subtype had the highest mean DFS (94.60 months) (Table 2) (Figure 1). DFS rates were significantly superior (P=0.002) in patients with luminal A type than in those with luminal B type breast cancer, with a median DFS of 59.32 and 40.03 months, respectively (Table 3).

**Table 2:** survival outcome according to biologic subtypes

Molecular subtype	Mean DFS (months)	SE	P=0.023
Luminal A	59,329	2,227	
Luminal B	40,036	2,632	
HER2+	89,054	7,539	
TNBC	94,608	6,222	
Overall	90,464	5,964	

DFS: disease free survival; SE: standard error; HER2: human epidermal growth factor receptor 2; TNBC: triple negative breast cancer

**Table 3:** comparison of disease free survival rates among breast cancer groups

Groups	P value
Luminal A vs luminal B	0.002
Luminal A vs HER2 +	0.097
Luminal B vs TNBC	0.035
HER2+ vs TNBC	0.494

## Discussion

It has been well documented that young age is as an unfavourable independent factor in prognosis and the involvement of risk factors and biopathological prognostic factors in the poorer prognosis of this subset of patients have been previously reported [6-9]. The present study is the unique and the largest study from Algeria to date showing the association of prognostic features and the prevalence of molecular phenotypes with regard BC patient´s age groups at diagnosis. From the Surveillance, Epidemiology, and End Results (SEER) database, median age of female BC at diagnosis was 61.0 [10], while the median age at presentation in our study is around 50 years, as well as other developing Arab countries [11].

It has been determined that an advance stage of presentation involving a large tumor size, a lymph nodes positivity, a high grade of differentiation lower HR expression and presence of triple negativity contributes in poor outcome in young patients [12]. We noted as is reported in literature [13,14] that the majority of patients presented with tumor size at T2 and a lower patients presented with SBR stage I, which is an indicator of the late coming of women for diagnosis. Approximately 5-10% of patients with breast cancer are diagnosed with distant metastasis at initial presentation [15], this statement is in concordance with our findings which informs us about the mostly pejorative prognosis of our patients.

The distribution of the various prognostic factors did not differ significatively in the age groups except for histological types, hormone receptors positivity and molecular phenotypes. Indeed through analyzing the clinicopathological trends of our cohort, a high incidence of ductal carcinomas was similary found among young and middle aged patients but less in aged patients, whereas, a minority of patients were diagnosed at SBR grade I, this proves that there is a real problem of breast cancer screening among this category of women. Lymph node metastasis is an important prognostic factor, indicating the advanced status of the disease and the likelihood that cancer cells have spread to distant sites, we noted that the proportion of their presence was higher than in Moroccan patients [16].

Hormonal analysis and molecular subtyping are used as an important predictive and prognostic factors in women with carcinoma of the breast. The presence of hormone receptors is correlated to a favourable prognosis, when young BC women were estrogen receptor positive and progesterone receptor negative they have a poor prognosis [17]. A high percentage of hormone receptor positivity was noted in this cohort, this correlates with the recently reported study from north India [9] and for our surprise and in contrast with other studies [18,19] their expression seems to decrease with age i.e. 66.9% (<40 years) versus 62.3% (44-54 years), versus 56.5% (≥55 years).

Carcinoma of the breast in young women shows variation in the prevalence of molecular subtypes in different regions of the world [20]. At a molecular level, data correlates with those of Znati *et al*. [16] and those of Tang *et al*. [21], where, luminal A comes in the first rank, this statement is different from studies belonging to African or American ethnicity where HER2, triple negative and luminal B subtypes seems to be more common among young patients compared to luminal A, indicating aggressive disease, higher grade, and poorer prognosis [19-22]. This distinction is important because HR+/HER2- (luminal A) is associated with good prognosis than HR+/HER2+ (luminal B) and triple negative type, several studies have showed that differences in biologic subtypes may vary by race as a function of age [22].

We noted a high prevalence of triple negative tumors in our study among patients above 55 years, the reasons remain unknown, however, we can supposed that our patients may represent a BRCA 1 mutations, since this gene alteration is associated with this molecular phenotype [23], furthermore, we can explain it by the fact that Algerian patients are for African ethnicity found to be more affected by this breast cancer phenotype among aged women [22]. Patients who express HER2 protein have less overall survival than those who do not being an aggressive factor of poor prognosis; the HER2-positive proportion in our cases is two fold high to that describe in Indian subcontinent and western literature [19], suggesting a poor prognosis and outcome profile.

To our surprise, it is the TNBC tumors that showed the most favorable DFS time compared to the other biological groups while TNBC tumors are known to be aggressive with high grade and for having the capacity to develop metastases. Our finding also reflects a better luminal A DFS rates than for luminal B patients, indeed, studies have shown that luminal A mortality rates were constant over time with mortality rates of luminal B HER2-positive and non-luminal subtypes tending to peak within 5 years after diagnosis which then declined over time [24,25].

## Conclusion

We report that younger patients had a higher percentage of positive hormone receptors than expected and were luminal A in contrast of aged patients who were TNBC molecular phenotype with the best DFS survival. This can revive us to other perspectives, that of understanding the tumor behavior of our population and of focusing the study on the potential relationship between hormone dependent patients and prognostic factors.

### What is known about this topic

- Breast cancer arising at a young age, it is agressive, have a poorer outcome than in elder women and is uncommon, particularly in the developed world;
- Younger women with breast cancer are more likely to develop more aggressive subtypes, which include a higher proportion of basal-like and HER2 over-expressing tumors that are associated with a poor prognosis;
- At the same time, we have noted that in women with HER2 over expression or triple negative breast cancer, the risk of recurrence seems to be similar in younger women compared with older women.

### What this study adds

- This study is unique in Algeria conducted with the largest cohort of breast cancer patients to have the most informative data on the state of this cancer in Algeria, in fact few studies are published by epidemiologists who generally only establish a national cancer registry;
- Multiple studies have attempted to answer the question of the role of age on breast cancer prognosis, taking into account factors of poor prognosis such as stage, histological grade and hormone receptor. The current study conducted on a large cohort of cancer patients aimed to explore for the first time this problem in Algeria, as it has been done before in other north African countries. This led us to observe that young women are good prognosis compared to their elders, this finding is not common to several studies;
- In addition, our study have found that patients with triple negative breast cancer molecular subtype had an exceptional good disease free survival, which surprised us. We think that breast cancer behave differently in this region of Algeria which implies more investigations into the reasons.
